# Obesity due to MC4R deficiency is associated with reduced cholesterol, triglycerides and cardiovascular disease risk

**DOI:** 10.1038/s41591-025-03976-1

**Published:** 2025-10-16

**Authors:** Stefanie Zorn, Rebecca Bounds, Alice Williamson, Katherine Lawler, Ruth Hanssen, Julia Keogh, Elana Henning, Miriam Smith, Barbara A. Fielding, A. Margot Umpleby, Summaira Yasmeen, Maria Marti-Solano, Claudia Langenberg, Martin Wabitsch, Tinh-Hai Collet, I. Sadaf Farooqi

**Affiliations:** 1 University of Cambridge Metabolic Research Laboratories and NIHR Cambridge Biomedical Research Centre, Institute of Metabolic Science, University of Cambridge, Cambridge, UK; 2 Division of Pediatric Endocrinology and Diabetes, Center for Rare Endocrine Diseases, Department of Pediatrics and Adolescent Medicine, Ulm University Medical Center and German Center for Child and Adolescent Health (DZKJ), Ulm, Germany; 3 Precision Healthcare University Research Institute, Queen Mary University of London, London, UK; 4 Computational Medicine, Berlin Institute of Health at Charité-Universitätsmedizin Berlin, Berlin, Germany; 5 Max Planck Institute for Metabolism Research, Cologne, Germany; 6 Faculty of Medicine and University Hospital Cologne, Polyclinic for Endocrinology, Diabetology and Preventive Medicine (PEPD), University of Cologne, Cologne, Germany; 7 Faculty of Health & Medical Sciences, University of Surrey, Guildford, UK; 8 Department of Pharmacology, University of Cambridge, Cambridge, UK; 9 Service of Endocrinology, Diabetology and Metabolism, Department of Medicine, Geneva University Hospitals, Geneva, Switzerland; 10 Diabetes Centre, Faculty of Medicine, University of Geneva, Geneva, Switzerland

**Keywords:** Endocrine system and metabolic diseases, Metabolism, Genetic association study

## Abstract

Obesity causes dyslipidemia and is a major risk factor for cardiovascular disease. However, the mechanisms coupling weight gain and lipid metabolism are poorly understood. Brain melanocortin 4 receptors (MC4Rs) regulate body weight and lipid metabolism in mice, but the relevance of these findings to humans is unclear. Here we investigated lipid levels in men and women with obesity due to MC4R deficiency. Among 7,719 people from the Genetics of Obesity Study cohort, we identified 316 probands and 144 adult family members with loss-of-function (LoF) *MC4R* mutations. Adults with MC4R deficiency had lower levels of total and low-density lipoprotein (LDL)-cholesterol and triglycerides than 336,728 controls from the UK Biobank, after adjusting for adiposity. Carriers of LoF *MC4R* variants within the UK Biobank had lower lipid levels and a lower risk of cardiovascular disease, after accounting for body weight, compared to noncarriers. After a high-fat meal, the postprandial rise in triglyceride-rich lipoproteins and metabolomic markers of fatty acid oxidation were reduced in people with MC4R deficiency compared to controls, changes that favor triglyceride storage in adipose tissue. We concluded that central MC4Rs regulate lipid metabolism and cardiovascular disease risk in humans, highlighting potential therapeutic approaches for cardiovascular risk reduction.

## Abstract (web summary)

The authors explore the impact of MC4R deficiency on lipid metabolism in humans, based on two large cohorts. Findings indicate that individuals with obesity due to MC4R deficiency have lower cholesterol and triglyceride levels, as well as reduced cardiovascular risk.

## Main

Environmental, social and genetic factors promote excess food intake and reduced physical activity, to drive weight gain^1^. Over time, increased adiposity is commonly associated with the development of hypertension, dyslipidemia and cardiovascular disease^2,3^. Obesity-associated dyslipidemia is characterized by elevated levels of serum triglycerides (TGs), high levels of low-density lipoprotein (LDL)-cholesterol and low levels of high-density lipoprotein (HDL)-cholesterol, a profile that is proatherogenic^4–6^. However, the mechanisms by which obesity drives changes in lipid levels are poorly understood.

Although research into the absorption, synthesis and distribution of lipids has focused on the role of the gut and liver, there is increasing recognition that the brain plays a key role in regulating peripheral lipid metabolism^7^. Most attention has focused on the leptin–melanocortin pathway^8,9^ which plays a critical role in maintaining body weight and coupling changes in nutritional state to physiological functions such as growth, pubertal timing and immunity^10^. Fasting or nutritional insufficiency causes a fall in circulating leptin levels, triggering a set of adaptive responses to restore energy homeostasis and defend against starvation^11^. Leptin’s effects are mediated in part by hypothalamic neurons expressing melanocortin 4 receptors (MC4Rs)^12^, which synapse with preganglionic sympathetic neurons projecting to the vasculature, liver and adipose tissue^13,14^. In mice, experiments using MC4R agonists and antagonists have shown that central melanocortin signaling regulates glucose uptake, TG synthesis, lipid deposition, cholesterol levels and lipid mobilization, in part by altering sympathetic nervous system (SNS) tone^15,16^. To date, the relevance of these findings to human lipid metabolism is unknown.

Multiple susceptibility alleles contribute to a person’s propensity to gain weight or stay thin in an obesogenic environment^17,18^. These genetic influences can be captured using a polygenic risk score (PGS) in people with common obesity^19^. In addition, some people harbor rare, more penetrant genetic variants that drive their obesity. Heterozygous loss-of-function (LoF) variants in *MC4R* represent the most common penetrant genetic form of obesity described to date^20–22^. Here to investigate the possible role of MC4R in human lipid metabolism, we studied a clinically ascertained cohort of people with severe obesity in whom variants in *MC4R* were identified. To test whether our findings can be replicated independently, we studied *MC4R* variant carriers in a large population-based cohort, UK Biobank. In a clinical study, we studied the postprandial response to a high-fat meal in people with MC4R deficiency and age-matched and body mass index (BMI)-matched control individuals.

## Results

### Defining a cohort of people with MC4R deficiency

We sequenced 7,719 children with severe early onset obesity (defined as a BMI standard deviation score (SDS) >3; onset before age 10 years) recruited to the Genetics of Obesity Study (GOOS) cohort (www.goos.org.uk). Here we collated data on coding variants in *MC4R*, some of which have been reported previously^20,21,23^. Variants were classified as pathogenic if they impaired one or more molecular mechanisms in cells, including trafficking to the cell membrane, signaling, binding or dimerization^24,25^, and if there was co-segregation of the variant with obesity in families (Supplementary Table 1).

**Fig. 1:**
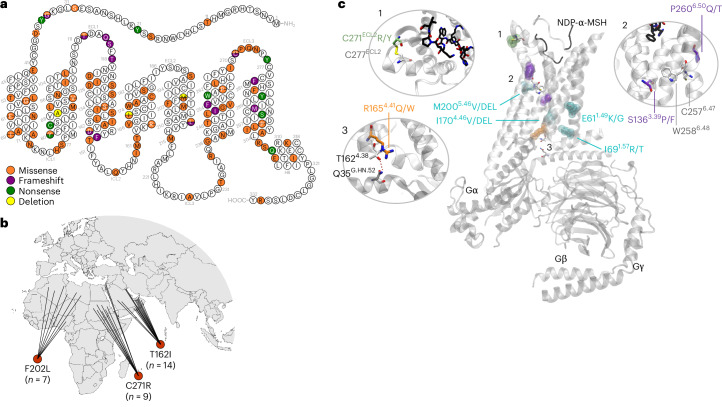
Genetic variants in *MC4R* in the GOOS cohort. **a**, Schematic of the human MC4R protein, a seven transmembrane domain, G-protein-coupled receptor. Three ECLs, three ICLs and a C-terminal helix 8 (H8) are indicated. Genetic variants affecting MC4R identified in the GOOS cohort are shown using a color code to indicate amino acid change and type of mutation(s) affecting the residue. Details of all variants are found in Supplementary Table 1. **b**, Some *MC4R* variants more prevalent within certain ethnic groups (countries of origin and number of probands in the GOOS cohort shown). **c**, Structural mapping of pathogenic variants into an agonist-bound MC4R structure. There is a general view of MC4R (white) in complex with a heterotrimeric Gs protein (silver) and the receptor agonist (Nle4, d-Phe7)-α-melanocyte-stimulating hormone (NDP-α-MSH; black). Mutated residues are highlighted depending on their predicted alteration of ligand-binding (green), receptor activation switches (purple), G-protein interaction (orange) or receptor homodimerization (cyan). Insets provide further structural detail of the residue or residue contacts that may be lost on mutation, with receptor and G-protein residues annotated using GPCRdb and G-protein common numbering schemes, respectively (Methods).

Of the 7,719 probands screened for *MC4R* variants, we found that 316 had pathogenic variants, giving a prevalence of 4.1% in this cohort. Although most probands carried heterozygous variants (*n* = 248), we also identified 65 probands with biallelic variants (56 with homozygous variants, 9 with different *MC4R* variants on each allele) (Fig. 1a and Supplementary Table 1). Some mutations appear to be more prevalent in certain ethnic groups, suggesting a founder effect, for example, Thr162Ile and Cys271Arg were found in multiple unrelated probands of Arab ancestry and Phe202Leu was found in multiple unrelated children of African ancestry (Fig. 1b and Supplementary Table 1).

Some variants were found in multiple unrelated probands of European ancestry: Tyr35Ter; Asp37Val (*n* = 24), Phe280AlafsX12 (*n* = 11), Ile125Lys (*n* = 19), Cys271Tyr (*n* = 13) and Glu61Lys (*n* = 11) (Supplementary Table 1). Some of these variants occur at multiallelic sites (for example, Arg165Gln (*n* = 7) or Arg165Trp (*n* = 11)) which may represent mutational hotspots. Crystal structures of MC4R in the inactive and active state^26–29^ are now available, allowing interrogation of the mechanisms by which pathogenic mutations cause LoF of the receptor. Notably, pathogenic mutations are distributed along the MC4R protein (Fig. 1a) and have the potential to disrupt structural hotspots, which regulate specific molecular mechanisms involved in signal transduction. These include Cys271Arg or Cys271Tyr (Fig. 1c) in the receptor’s extracellular loop 2 (ECL2), which eliminates a disulfide bridge with neighboring Cys277 and could thus alter the integrity of the ligand-binding site. Mutations in and around the highly conserved CWxP motif found in the core of class A G-protein-coupled receptors (GPCRs), including P260^6.50^Q/T and S136^3.39^P/F (Fig. 1c), are predicted to compromise the toggle-like movement of transmembrane helix 6 (TM6) that is associated with receptor activation across GPCRs^29,30^. In addition, variants such as R165^4.41^Q/W (Fig. 1c), which are in close proximity to the G-protein coupling interface, could alter signal transduction by destabilizing the interaction between T162^4.38^ at the interface between TM4 and intracellular loop 2 (ICL2) of the receptor and residue Q35^G.HN.52^ in the αN-helix of Gαs. A final group of mutations found in transmembrane helices TM1, TM4 and TM5 (Fig. 1c) may alter two previously described MC4R homodimer interfaces between TM1 and TM7 and between TM4 and TM5. Indeed, our previous characterization of missense variants in these interfaces, which included the M200^5.46^V mutant, revealed decreased dimerization with wild-type MC4R^24^.

**Extended Data Fig. 1:**
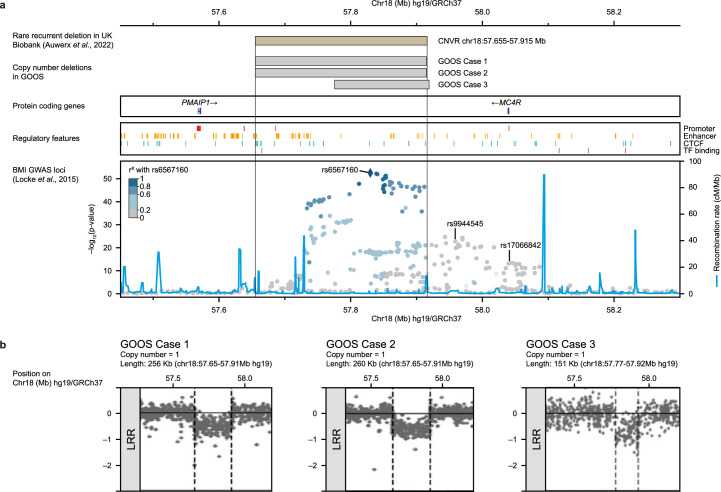
Copy number variants encompassing the MC4R gene. **(a)** A rare recurrent deletion (copy number variable region (CNVR) hg19/GRCh37 chr18:57,655-57,915 Kb; length=260 Kb) found in three probands in the GOOS cohort and previously studied in UK Biobank^32^. The genomic region is annotated with protein-coding genes, regulatory regions (Ensembl GRCh37 release 112) and BMI-associated GWAS loci^88^. The points labelled by rsID indicate the three conditionally independent SNPs previously reported by Locke et al.^83^. The LocusZoom visualisation was obtained at https://my.locuszoom.org (ref. ^89^). **(b)** Log-R ratio (LRR) plots of the three hemizygous deletion calls in GOOS probands (GRCh37/hg19).

As deletions on chromosome 18 affecting *MC4R* have been reported, we ascertained the frequency of copy-number variants (CNVs) involving the 18q21.32 chromosomal region in 1,386 GOOS participants of European ancestry (Methods). We found that two people had a 260-kb hemizygous deletion (chr18:57.65-57.91Mb) and one a smaller 151-kb deletion with a similar distal breakpoint (chr18:57.77-57.92Mb). These recurrent hemizygous deletions involve a genome-wide association study locus for BMI near the *MC4R* gene (Extended Data Fig. 1)^31^. The rare recurrent hemizygous 260-kb deletion (nearest gene, *MC4R*; *n* = 46 of 331,572; ~0.014%) has been associated with BMI in UK Biobank^32^. Among UK Biobank participants with this deletion, 48% of deletion carriers self-report being ‘plumper at age 10’ compared to 22.9% of BMI-matched noncarriers (Supplementary Table 2; *P* = 1.73 × 10^−5^) and 16% of UK Biobank participants. Deletion carriers were excluded from subsequent analyses.

### Clinical features in adults with MC4R deficiency

**Fig. 2:**
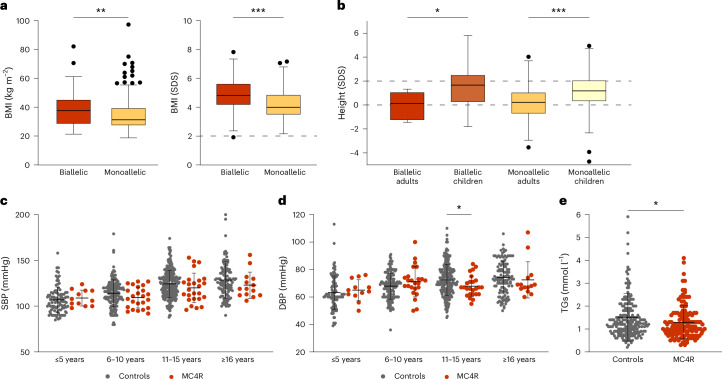
Clinical phenotypes associated with MC4R deficiency. **a**, BMI (median difference −3.98, 95% CI −7.00 to −1.00, *P* = 0.007) (left) and BMI SDS (median difference −0.69, 95% CI −0.98 to −0.40, *P* = 0.001) (right). **b**, Height SDS of people with biallelic (*n* = 63, median difference 1.60, 95% CI 0.52–2.74, *P* = 0.007) and monoallelic (*n* = 274, median difference 1.00, 95% CI 0.79–1.23, *P* = 0.001) *MC4R* variants in the GOOS cohort. The box plots illustrate the distribution of data points, showing median and interquartile range (IQR), whereas the boxplot whiskers represent quartile 1 (Q1) − 1.5× IQR and Q3 + 1.5× IQR. **c**,**d**, Systolic (SBP; **c**) and diastolic (DBP; **d**) blood pressure by age group of people with MC4R deficiency (*n* = 72, red) and age-matched and BMI-matched control individuals (*n* = 574, gray) in the GOOS cohort. The mean ± s.d. values are shown; there is a significant difference in diastolic blood pressure at age 11–15 years (median difference 4.0, 95% CI 0.0–8.0, *P* = 0.04). **e**, Fasting serum TG concentrations in people with MC4R deficiency (*n* = 131, red) and age-matched and BMI-matched control individuals (*n* = 211, gray) in the GOOS cohort. The mean ± s.d. values are shown: median difference −0.1, 95% CI −0.3 to 0.0, *P* = 0.04. The statistical significance in each panel was determined using the two-tailed Mann–Whitney *U*-test. ^*^P < 0.05, ^**^P < 0.01, ^***^P < 0.001.

**Extended Data Fig. 2:**
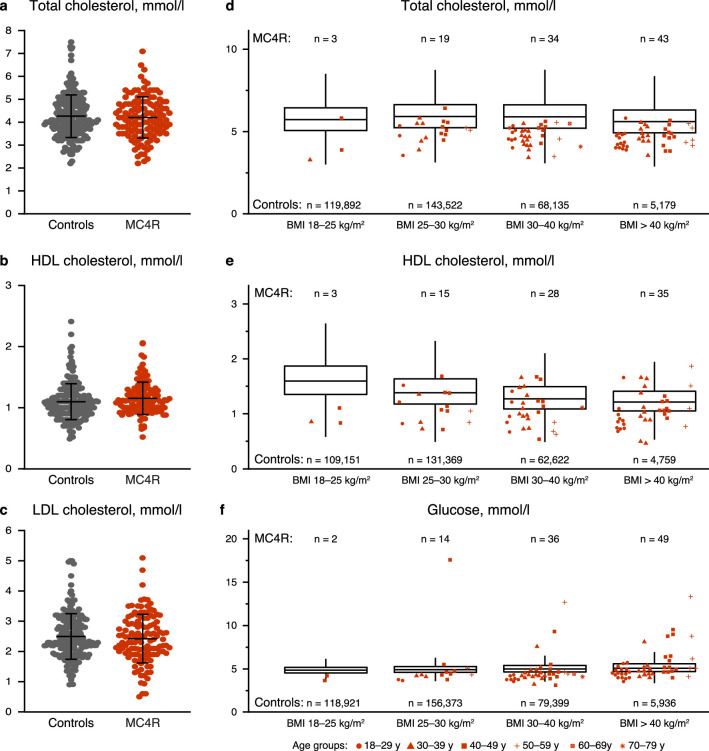
Cardiometabolic phenotypes in people with MC4R deficiency from GOOS compared to control individuals in GOOS and the UK Biobank. **(a)** Fasting total cholesterol, **(b)** HDL-cholesterol and **(c)** LDL-cholesterol levels in people with MC4R deficiency (red; n = 131) and age and BMI matched controls (grey; n = 211) from the GOOS cohort; mean ( ± SD) values are shown. **(d)** Total cholesterol, **(e)** HDL-cholesterol and **(f)** random glucose concentrations in loss of function *MC4R* variant carriers compared to non-carrier control individuals in the UK Biobank, stratified by BMI category. Points (red) represent individual *MC4R* variant carriers, with age groups represented by point shape. The boxplot whiskers represent Q1-1.5 (interquartile range) and Q3 + 1.5 (interquartile range) in UK Biobank. The number of *MC4R* variant carriers (top) and UK Biobank controls (bottom) are shown for each comparison group.

Family co-segregation studies of the 316 GOOS probands with pathogenic variants in *MC4R* identified a further 461 people with MC4R deficiency (Supplementary Table 1). In studies of 777 people with MC4R deficiency (the largest cohort reported to date), we found that the severity of obesity is influenced by gene dosage, because people with biallelic mutations have a higher BMI and BMI SDS than those with monoallelic or heterozygous mutations (Fig. 2a) and that penetrance is highly variable. Previous studies have described the clinical features of MC4R deficiency as hyperphagia (increased drive to eat), weight gain in the first 5 years of life, disproportionate hyperinsulinemia and accelerated linear growth in childhood^20,33^. Here we found that, although height SDS is relatively increased in children with monoallelic and biallelic *MC4R* mutations, final adult height is within the normal range in both groups (Fig. 2b). We found that adolescents with MC4R deficiency had lower diastolic blood pressures compared to control individuals in the GOOS cohort without mutations in known obesity genes matched for age and BMI (Fig. 2c,d), in keeping with previous studies in adults by ourselves and others^34,35^. Individuals with MC4R deficiency also had lower fasting TG concentrations (Fig. 2e), although the total serum cholesterol, LDL-cholesterol and HDL-cholesterol levels were comparable to those seen in age-matched and BMI-matched control individuals (Extended Data Fig. 2a–c).

**Fig. 3:**
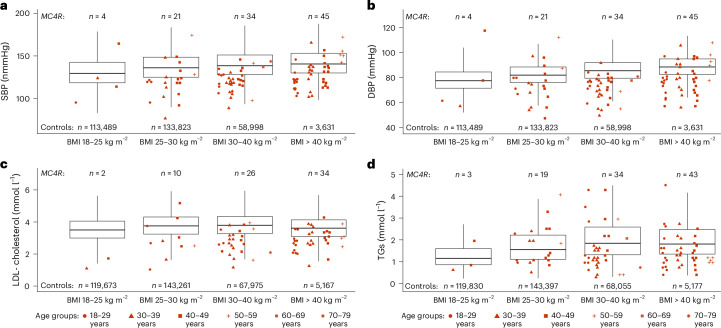
Cardiometabolic traits in *MC4R* variant carriers in GOOS compared to control individuals in the UK Biobank. **a**–**d**, Comparison of systolic blood pressure (**a**), diastolic blood pressure (**b**), LDL-cholesterol (**c**) and TGs (**d**) in adult carriers of pathogenic *MC4R* variants in GOOS compared to noncarrier control individuals in the UK Biobank, stratified by BMI category. Points represent individual *MC4R* variant carriers (red), with age groups of these individuals represented by point shape. The boxplot whiskers represent Q1 − 1.5× IQR and Q3 + 1.5× IQR in the UK Biobank. The number of GOOS *MC4R* variant carriers (top) and UK Biobank controls (bottom) are shown for each comparison group.

Next, we compared adults with pathogenic *MC4R* mutations in GOOS to a large-scale, population-based control dataset, the UK Biobank study (Methods; maximum *N* = 336,728 males and females not carrying *MC4R* variants). We found that adults in the GOOS cohort with pathogenic *MC4R* mutations (*n* = 144) had lower systolic and diastolic blood pressures compared with non-*MC4R* variant carriers in UK Biobank at every level of BMI (Fig. 3a,b and Supplementary Table 3). In addition, GOOS *MC4R* variant carriers had lower levels of total cholesterol (Extended Data Fig. 2d), LDL-cholesterol (Fig. 3c) and TGs (Fig. 3d), but not HDL-cholesterol or blood glucose levels after adjusting for the BMI (Extended Data Fig. 2e,f and Supplementary Table 3). Although adults in the GOOS were younger than their UK Biobank counterparts, differences in age had no major impact on these findings (Fig. 3 and Extended Data Fig. 2).

### Study of *MC4R* variant carriers in the UK Biobank

We recognized that recruitment criteria, methods of clinical measurement and biochemical assays differed between the GOOS and the UK Biobank. To test whether our findings replicate for pathogenic *MC4R* variants observed in the large, unselected, population-based UK Biobank study, we tested whether LoF *MC4R* variants (identified in whole-exome sequencing (WES) data) were associated with cardiometabolic traits and disease in European ancestry individuals in the UK Biobank, using gene burden analyses. *MC4R* variants were identified in up to 42,761 individuals. A subset of the *MC4R* variants identified in the UK Biobank (78 of 186; 41%) were defined as LoF if they had been shown to significantly impair function of one or more molecular mechanisms in cell-based assays (Methods and Supplementary Table 4). We found that carriers of LoF *MC4R* variants had significantly lower total cholesterol, LDL-cholesterol and TG levels compared to noncarriers after adjusting for BMI (Supplementary Table 5). The UK Biobank *MC4R* variant carriers also had a lower risk of hypertension for a given BMI compared to noncarriers and lower self-reported use of blood pressure-lowering medication (Supplementary Table 5). A directionally consistent trend of association was observed for lower systolic and diastolic blood pressure in *MC4R* variant carriers; however, these did not meet statistical significance (Supplementary Table 5).

Given the protective associations with risk factors for cardiovascular disease (CVD), we investigated associations of *MC4R* variants with a selected set of CVD outcomes (Methods). LoF *MC4R* variants were not significantly associated with any CVD measure (odds ratio (OR) range 0.997–1.034), before or after adjusting for the BMI (Supplementary Table 6). In contrast, individuals with a high PGS for BMI (that is, common obesity) showed a significantly increased risk of CVD (OR range 1.22–1.38; *P* < 0.05). Furthermore, comparing the deciles of polygenic risk for obesity, those with the highest polygenic risk (decile 10) had a 17.5% increased risk of developing coronary artery disease compared to the lowest decile (OR = 1.175 (95% confidence interval (CI) 1.119–1.234), *P* = 1.05 × 10^−10^) and 11.5% increased risk compared to deciles 5 and 6 (OR = 1.116 (1.070–1.163), *P* = 2.64 × 10^−7^) as well as an increased risk of multiple obesity-associated conditions (Supplementary Tables 7–9).

**Extended Data Fig. 3:**
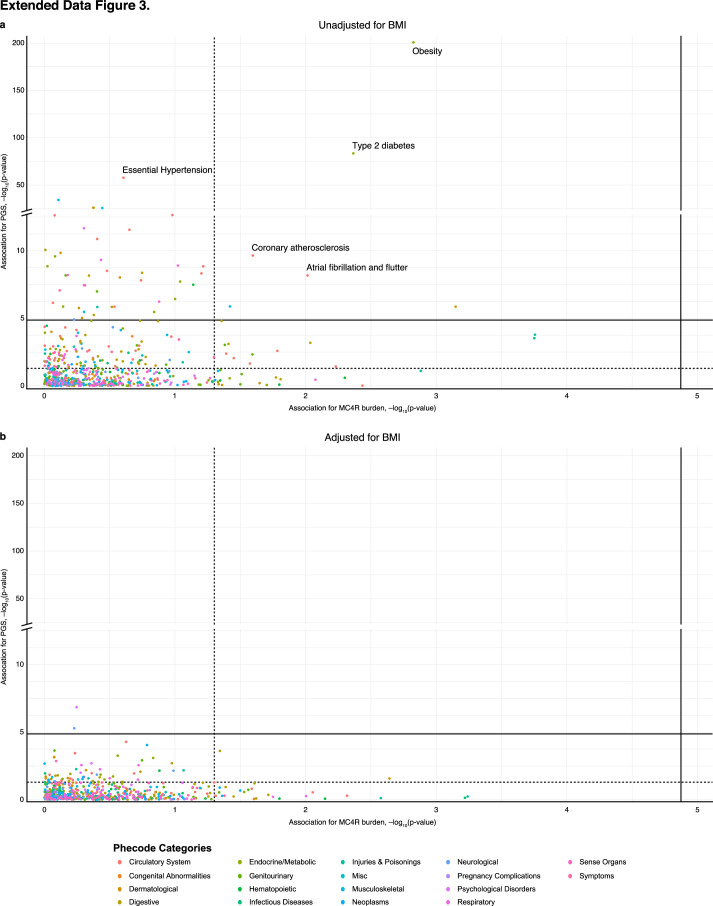
Phenome wide association study (PheWAS) for MC4R variant carriers and obesity polygenic score in the UK Biobank. Association analyses for MC4R LOF variants and Obesity (BMI) polygenic score (PGS) across disease outcomes as defined by phecodes in European ancestry individuals in the UK Biobank**. (a)**. PheWAS results from BMI unadjusted analyses and **(b)** BMI adjusted analyses; colour of the points represents phecode category (see legend). X-axis shows -log_10_(p-value) of association for MC4R LOF variants in gene burden analyses and Y-axis -log_10_(p-value) of association for obesity PGS. Dotted grey line represents nominal significance threshold (P < 0.05) and solid grey line represents Bonferroni significance (P < 1.34×10^−5^). Labels indicate cardiovascular system or endocrine/metabolic outcomes that are at least nominally (P < 0.05) associated with MC4R variants and significantly associated with the obesity PGS (P < 1.34×10^−5^). Additional points are labelled which are significantly associated with the obesity PGS but not MC4R burden. Y axis has been split between a -log_10_(p-value) of 15 and 30.

To investigate the potentially wider clinical impact of LoF *MC4R* variants, we conducted hypothesis-free gene burden analyses for 626 different diseases^36^ with a minimum number of 100 cases^36^. With the exception of obesity (*P* < 1.34 ×10^−5^), LoF *MC4R* variants were not significantly associated with any other condition or disease after correction for multiple testing (Supplementary Tables 10 and 11 and Extended Data Fig. 3).

In this study, we replicated the finding that carriers of two gain-of-function (GoF) MC4R variants (Val103Ile and Ile251Leu) had a lower BMI^25^ and also found that they had a favorable metabolic profile characterized by higher HDL-cholesterol, lower TGs and lower glycated hemoglobin; we did not observe any significant impact of these GoF variants on LDL-cholesterol levels, blood pressure or coronary artery disease risk in either BMI-unadjusted or BMI-adjusted models (Supplementary Tables 12 and 13). These findings support those from a previous study which found that heterozygous carriers of Val103Ile MC4R had lower TG levels compared to wild-type allele carriers^37^. Notably, Val103Ile and Ile251Leu MC4Rs were associated with a lower risk of myocardial infarction in BMI-adjusted and BMI-unadjusted models (Supplementary Tables 12 and 13).

Cumulatively, these studies in people from the GOOS and UK Biobank demonstrate that obesity due to MC4R deficiency is associated with reduced total cholesterol, LDL-cholesterol, TGs and hypertension, and relative protection from CVD.

### Postprandial lipid metabolism in MC4R deficiency

**Fig. 4:**
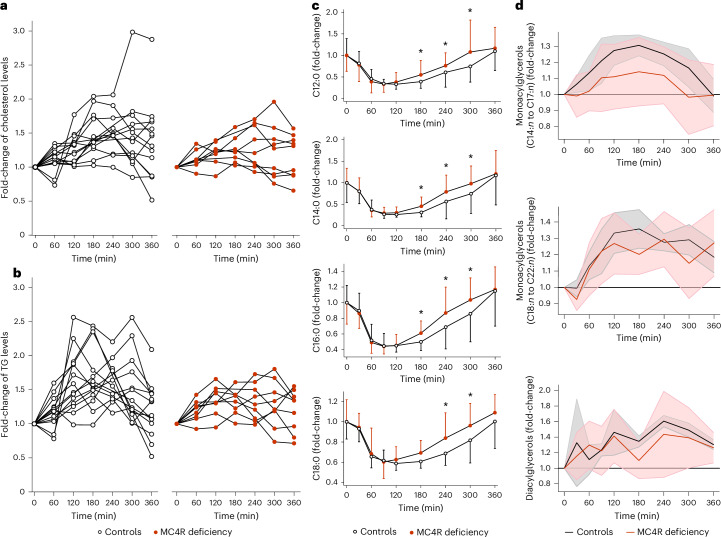
Postprandial lipids after a high-fat meal challenge. Lipid and lipoprotein levels before and after a high-fat meal challenge over 360 min in adults with MC4R deficiency (red solid circles) compared to age-matched, sex-matched and BMI-matched control individuals (empty circles). **a**,**b**, The fold-change in cholesterol (**a**) and TGs (**b**) in TRLs, relative to baseline values where each line and dot series represents an individual trajectory (*n* = 9 adults with MC4R deficiency (right), *n* = 14 controls (left)). **c**,**d**, Data from untargeted metabolomic analysis. **c**, Mean ± s.d. for free fatty acid concentrations of laurate (C12:0), myristate (C14:0), palmitate (C16:0) and stearate (C18:0). The two-sided statistical significance was determined using two-way ANOVA with repeated measures (*n* = 9 adults with MC4R deficiency, *n* = 10 controls). ^*^P < 0.05 (Supplementary Table 15). **d**, Monoacylglycerols and diacylglycerols (solid line, mean fold-change compared to baseline; s.d. range in shaded area). Top, monoacylglycerols (C14:*n*) to (C17:*n*). Middle, monoacylglycerols (C18:*n*) to (C22:*n*). Bottom, diacylglycerols.

**Extended Data Fig. 4:**
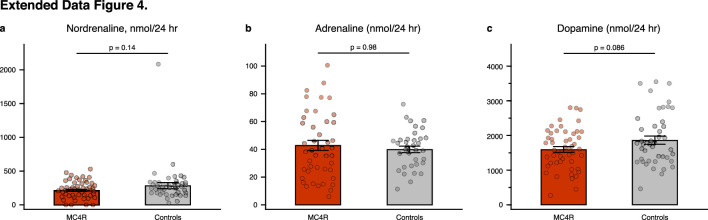
Urinary catecholamines in adults with MC4R deficiency and control individuals with obesity. **(a)** Noradrenaline, **(b)** adrenaline and **(c)** dopamine excretion in urine over 24 h in adults with MC4R deficiency (red; n = 50) and BMI matched control individuals (grey; n = 44) including people in the lipid study; mean ( ± SD) values are shown. Statistical significance tested using the Mann-Whitney-Wilcoxon ranksum test.

Humans spend most of their time in the postprandial state and studies have shown that postprandial TG levels predict CVD risk, potentially more so than fasting TG levels^38–40^. We therefore studied the postprandial response to dietary lipids using a high-fat meal challenge in 11 individuals with MC4R deficiency and 15 control individuals matched for age, sex, BMI and levels of fasting TGs and fasting plasma insulin (Supplementary Table 14). After a supervised overnight fast, samples were obtained before, and every 30 min after, the consumption of a high-fat meal (674 kcal; 60% fat) for 6 h. Fasting very-low-density lipoproteins (VLDLs), intermediate-density lipoproteins and LDL content in TG, cholesterol and apolipoprotein-B 100 (apo-B100) and urinary catecholamines did not differ significantly between individuals with MC4R deficiency and controls (Supplementary Table 14 and Extended Data Fig. 4). Ingestion of the high-fat meal was associated with an increase of cholesterol and TGs in the TG-rich lipoprotein (TRL) fraction in controls with obesity. This excursion was significantly reduced in those with MC4R deficiency (Fig. 4a,b): mean ± s.d. area under the curve (AUC) of the postprandial TG response was 92 ± 49 mmol l^−1^ min^−1^ in MC4R deficiency compared to 156 ± 90 mmol l^−1^ min^−1^ in control individuals (*P* = 0.049 after adjustment for age and BMI). In addition, the peak postprandial concentration of TGs in the TRL fraction was reduced by 50% in MC4R deficiency compared to controls (0.49 ± 0.14 mmol l^−1^ versus 0.85 ± 0.39 mmol l^−1^, *P* = 0.03; Fig. 4b). We noted that the peak postprandial concentration of apo-B100 in TRL occurred earlier in MC4R deficiency (mean ± s.d. 160 ± 134 min) compared to controls (296 ± 64 min, *P* = 0.004 in unadjusted analysis and *P* = 0.03 after adjustment for age and BMI). Together, these findings indicate that, after a high-fat meal, the clearance of TRL (chylomicrons and VLDLs) is increased in MC4R deficiency. This could be due to higher lipoprotein lipase-mediated lipolysis, as seen in some previous studies of people with obesity^41^.

To examine changes in fatty acids and other lipid species produced by lipolysis, we performed a nontargeted metabolomic analysis in serum samples obtained in the fasted state and every 30 min for 6 h postprandially (Supplementary Table 15). We found that specific medium-chain and long-chain fatty acids increased in the late postprandial period to a greater extent in people with MC4R deficiency compared to control individuals matched for age, sex and BMI (Fig. 4c and Supplementary Table 15), representing the appearance of meal-derived fatty acids^42^. This suggests that lipoprotein lipase-mediated lipolysis of TG contained within chylomicrons^43^ may be increased in MC4R deficiency. This feature has been proposed as being associated with a metabolically healthier phenotype^44^. In control individuals with obesity, the decrease in medium-chain and long-chain fatty acids from 60 min was followed by the accumulation of fatty acids and glycerol to form monoglycerols and diacylglycerols; the accumulation of monoglycerols and diacylglycerols was attenuated in MC4R deficiency (Fig. 4d and Supplementary Table 15). Together, these changes may result in a larger pool of nonesterified fatty acids available for oxidation in the postprandial period^45,46^. The decrease in acyl carnitines (products of fatty acid oxidation) in response to the high-fat meal challenge was reduced in MC4R deficiency (Supplementary Table 15), suggesting that fatty acid oxidation is impaired in people with MC4R deficiency.

## Discussion

We have studied the largest available cohort of people with MC4R deficiency, providing insights into the genetic and clinical spectrum of this disorder, the most common monogenic form of obesity. Previous studies have shown that the prevalence of LoF *MC4R* variants ranges from approximately 0.3% in an unselected birth cohort^47^ to 1% of people with a BMI > 30 kg m^−2^ (refs. ^25,48^) to 2–5% of children with obesity^21,49,50^. In the GOOS cohort, a clinically ascertained group of people with severe early onset obesity, we found the prevalence of LoF *MC4R* variants to be 4.1%. We identified a range of biallelic mutations in probands from consanguineous families (homozygous mutations), but also children who carried two different variants, each inherited from parents who were not related to each other but had obesity. Although biallelic variant carriers have a higher BMI than monoallelic or heterozygous carriers, there is considerable clinical heterogeneity. These findings suggest that additional modifying factors that affect the expression and/or function of MC4R are likely to contribute to variable penetrance. Although, to date, treatment options for people with severe obesity due to MC4R deficiency have been limited, recent evidence suggests that the dual GLP-1 and GIP receptor agonist, tirzepatide, is effective at inducing weight loss in this group (see the accompanying paper by Bhatnagar et al.^51^).

We found that 27.5% of the *MC4R* variants in GOOS were stop gains, frameshift or deletion variants, whereas 72.5% were missense variants (Supplementary Table 1). This mutational spectrum contrasts with that seen in the UK Biobank, where 91.5% of carriers had missense variants and only 8.5% carried stop gains, frameshift or deletion variants. Previous studies have highlighted that people recruited into population-based cohorts such as the UK Biobank are generally healthier^52^. Our findings support this assertion. As seen here for MC4R deficiency, differences in the number and type of genetic variant carried by people in population versus clinical cohorts can impact the outcome and effect size seen in association studies^52^.

Here we replicate earlier studies showing that blood pressure is reduced in people with obesity due to MC4R deficiency^34,35^, an effect that is likely to be driven by impaired SNS tone^34,35^. In two independent cohorts, one clinically ascertained, the other population derived, we demonstrated that MC4R deficiency is associated with significantly lower concentrations of total cholesterol, LDL-cholesterol and TGs, with no effect on HDL-cholesterol. It is interesting that this profile is comparable to that seen in people who undergo prolonged caloric restriction^53^. For example, a study of healthy volunteers with obesity undergoing a medically supervised fast for 14 days (250 kcal d^−1^) showed that levels of total cholesterol, LDL-cholesterol and total serum TGs were significantly reduced in that time frame^54^.

**Fig. 5:**
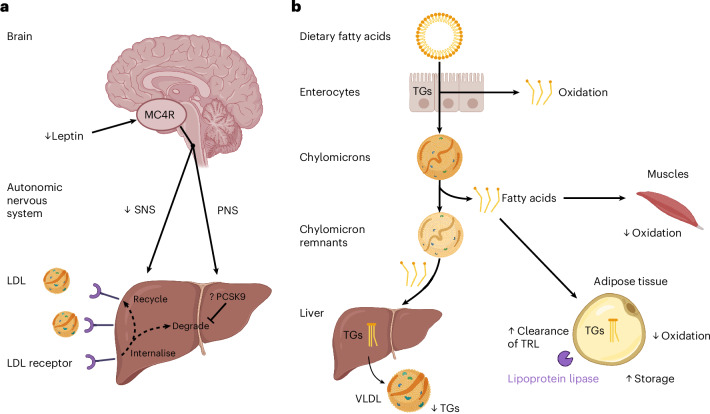
Model of changes in lipid metabolism seen in people with MC4R deficiency. **a**,**b**, A simplified schematic of human lipid metabolism showing key steps in the fasting (**a**) and postprandial (**b**) states. Changes seen in MC4R deficiency are indicated by arrows. **a**, In the fasting state, a fall in circulating leptin inhibiting signaling by hypothalamic neurons expressing MC4Rs. These neurons synapse with SNS neurons to regulate hepatic lipid mobilization. Fasting and MC4R deficiency impair SNS tone; changes in SNS tone are counterbalanced by changes in the parasympathetic nervous system. LDL-cholesterol is absorbed from the circulation by LDL receptors on hepatocytes. After binding LDL, LDL receptors are internalized and either recycled to the cell membrane or degraded by PCSK9. We hypothesize that, in MC4R deficiency (indicated by ‘?’), lower PCSK9 expression leads to increased LDL-receptor protein levels at the cell membrane and reduced serum LDL-cholesterol concentrations. **b**, In the postprandial state, dietary fatty acids absorbed by enterocytes either oxidized or conjugated into TGs, which, with lipoproteins, are absorbed as chylomicrons. In a series of steps, fatty acids are released for oxidation by skeletal muscle, recycled as TRLs, which are cleared by the liver or stored as TGs in adipose tissue. In MC4R deficiency (arrows), increased clearance of TRLs and reduced fatty acid oxidation favor increased TG storage in adipose tissue. PNS, peripheral nervous system; SNS, sympathetic nervous system. Panels **a** and **b** were created using BioRender.com.

How might fasting or nutritional insufficiency reduce LDL-cholesterol levels? Serum LDL-cholesterol levels are regulated by hepatic LDL receptors, which are critical for the clearance of lipids from the circulation and drive the uptake of cholesterol-containing lipoproteins^55^. Accordingly, LoF LDL-receptor mutations cause familial hypercholesterolemia and premature atherosclerosis^56^. LDL-receptor protein is degraded by proprotein convertase subtilisin or kexin type 9 (PCSK9): LoF mutations in PCSK9 protect against CVD, whereas GoF mutations are associated with hypercholesterolemia and accelerated CVD^57–59^, demonstrating the critical role of this pathway. Experiments in leptin-deficient *ob/ob* mice have shown that LDL-receptor protein (but not messenger RNA) levels are increased by approximately 60% (ref. ^60^); in addition, Pcsk9 expression is decreased in the livers of *ob/ob* mice^60^. We hypothesize that nutrient insufficiency (fasting, leptin deficiency, MC4R deficiency) leads to an increase in LDL-receptor protein levels, accelerating LDL-cholesterol clearance from the circulation to increase the availability of cholesterol for critical cellular functions (Fig. 5). This hypothesis, and the potential role of PCSK9 in regulating the effects of nutritional insufficiency on LDL-receptor protein levels, need to be tested in additional studies.

In the postprandial state, we found increased clearance of TRLs (chylomicrons and VLDLs) in MC4R deficiency, supported by the earlier peak in apoB-100 (a VLDL-specific protein) and by the metabolomic data. These changes would lead to increased TG storage in adipose tissue and lower liver fat. Although we did not measure liver fat in people in this study, we have previously shown that liver fat measured by magnetic resonance imaging is comparable in people with MC4R deficiency compared to age-matched and BMI-matched controls^35^. In addition, we did not see an increased prevalence of hepatic steatosis in the phenome-wide association study of people with LoF MC4R variants in the UK Biobank.

Our findings in humans do not align with findings in animals in a straightforward way^15,16^, which may be explained by species differences in lipid metabolism^40^. In mice, hypothalamic circuits regulate the metabolic switch from glucose to lipids and ketone bodies as a primary fuel during nutritional insufficiency^61^. Central administration of neuropeptide Y (released in the fasting state) increases hepatic VLDL–TG secretion^62^, resulting in the mobilization of lipids. Central administration of Melanotan-II, a synthetic MC3R and MC4R agonist, decreases hepatic TG content, whereas a MC3R and MC4R antagonist increases liver TG content^15^. Further studies using isotopic tracers are needed to quantify chylomicron and VLDL production and clearance rates in humans and to investigate whether the phenotype reported here may be the result of changes in intestinal and/or hepatic TG metabolism.

Studies involving both genetic ablation of β_1_-, β_2_- and β_3_-adrenergic receptors and denervation of sympathetic nerves in mice have shown that the effects of both neuropeptide Y and melanocortin peptides (acting via MC4R) on lipid metabolism are mediated by the autonomic nervous system^15,62^. As SNS tone is reduced in human MC4R deficiency^35^, we hypothesize that changes in lipid metabolism in humans are similarly mediated, at least in part, by reduced SNS tone^63^ (Fig. 5). Although SNS activation is highly conserved across species^64^, it is well known that multiple aspects of bile acid^65–67^ and lipid metabolism, including reverse cholesterol transport, differ between rodents and humans^68^. A particular example is cholesteryl ester transfer protein, which facilitates lipid transfer between lipoprotein pools, plays a key in HDL metabolism in humans but is absent in rodents^69^. In addition, there are species-specific differences in the effects of hormone action on lipid handling by the liver and adipose tissue^70^. We propose that these species-specific effects of MC4R deficiency on lipid metabolism may nevertheless be mediated by impaired SNS tone. This assertion requires further experimental validation in humans, for example, by directly investigating SNS innervation of adipose tissue.

Our study has several limitations. Although we have studied the largest available cohort of people with MC4R deficiency, the number of people included in the high-fat meal challenge is modest. To reduce heterogeneity, we matched people for age, sex, BMI, fasting TG and insulin levels (Methods). Additional studies of a larger cohort of people with MC4R deficiency are needed, using radioisotopes to quantify lipid species, precursors and metabolites, including measurements of flux and kinetics; direct measurement of lipoprotein lipase activity, which reflects the amount of lipoprotein lipase bound to endothelial sites (measured post-heparin administration), would also be useful. Given the direct effects of insulin on multiple aspects of lipid metabolism, cases and controls will need to be matched for insulin sensitivity. Previous studies using hyperinsulinemic euglycemic clamps have shown that insulin sensitivity and body fat distribution are comparable in adults with MC4R deficiency compared to age-matched, sex-matched and BMI-matched controls, but insulin secretion (measured by hyperglycemic clamps) may be increased^35^.

Our findings indicate that central melanocortin signaling is an important mechanism by which changes in body weight are coupled to changes in lipid metabolism. Previous cross-sectional and longitudinal epidemiological studies have shown that, as BMI increases, circulating levels of fasting TGs and total cholesterol also increase^71^. Reciprocally, weight loss reduces TG and cholesterol levels. However, finding the mechanisms that explain these effects has been challenging, in part due to the genetic and clinical heterogeneity of these common conditions. Penetrant genetic disorders such as MC4R deficiency, although relatively rare, can provide direct insights into the physiological role of specific molecular pathways. Indeed, in lipid metabolism, studies of patients with mutations in LDL receptors, PCSK9 and several other molecules have played a seminal role in revealing key regulators of lipid homeostasis, and the discovery and validation of targets for therapy (for example, statins and PCSK9 inhibitors^55,59^). Further investigation of the molecular and neural mechanisms that mediate the beneficial effects of MC4R inhibition on lipid metabolism are needed. Specifically, dissection of the SNS-mediated regulation of hepatic TG uptake, metabolism and secretion may provide new therapeutic targets for obesity-associated dyslipidemia and CVD.

## Methods

### Ethical approval and study design

All genetic and clinical studies were approved by the Multi-Regional Ethics Committee (no. 97/21) and the Cambridge Local Research Ethics Committee (no. 03/103) and conducted in accordance with the principles of the Declaration of Helsinki. Each participant provided written informed consent before inclusion in the study; children aged <16 years provided verbal assent. *MC4R* variant carriers were identified as part of the genetic studies of individuals recruited to the GOOS, a cohort of approximately 8,000 individuals with severe early onset obesity: age of obesity onset <10 years^20^. No statistical method was used to predetermine sample size. Severe obesity was defined as a BMI (weight in kilograms divided by the square of the height in meters) SDS ≥3 (SDS calculated according to the UK reference population). The functional properties of each *MC4R* variant were assigned based on published studies summarized on the website (www.mc4r.org.uk) and are included in Supplementary Table 1, with the baseline clinical features of variant carriers. Additional variant carriers were identified by conducting family co-segregation studies. For clinical analyses we excluded six people: two with a complete gene deletion, one with a heterozygous *MC4R* variant with unclear functional properties and three for whom the mode of inheritance was unclear due to the lack of parental samples.

Clinical and biochemical data were obtained from the medical records provided by physicians. For analysis of cardiometabolic traits, people with type 1 or 2 diabetes or known cardiometabolic or gastrointestinal diseases were excluded. Weight and height were measured barefoot in light clothing. Blood pressure was measured in the rested, fasted state using automated brachial (DINAMAP, GE Healthcare) or wrist (OMRON Healthcare) monitors. Blood samples were taken in the fasted state.

### MC4R structural analysis

Mapping of pathogenic variants was performed using the cryoelectron microscopy structure of the MC4R-Gs-NDP-α-MSH complex^29^ (Protein Data Bank, accession no. 7F53). Receptor and G-protein residues were annotated using GPCRdb^72^ and G-protein common numbering^73^ schemes, respectively. Structural visualization was performed using VMD v.1.9.4a57 (ref. ^74^).

### Statistical analyses and reproducibility

Detailed methods for each of the analyses, including design and statistical tests used, are presented below.

### CNV analysis of the GOOS from genotyping arrays

Affymetrix Human SNP Array 6.0 chip array data from 1,386 GOOS participants with self-reported white British ancestry were reviewed^75^. We called CNVs on chromosome 18 using R/Bioconductor crlmm (v.1.48.0) and VanillaICE (v.1.52.0)^76^. GC wave correction was performed using arrayTV (v.1.24.0).

### The 18q21.32 deletion carriers matched the cohort analysis

The UK Biobank study is a prospective population-based study of ~500,000 individuals recruited from across the United Kingdom^77^. These genetic analyses were conducted via the UK Biobank research analysis platform (RAP; application no. 53821, field names provided in brackets below). Comparison of GOOS participants with the UK Biobank was conducted using R (v.4.0.3). The visualization of results was performed using R package ggplot2 (v.3.3.5). Samples in the UK Biobank had been genotyped with either the Applied Biosystems UK Biobank Axiom Array or the Applied Biosystems UK BiLEVE Axiom Array by Affymetrix, which share 95% probe overlap^77^. The log(*R* ratio) and beta-allele frequency values were obtained from the UK Biobank (field nos. 22431 (v.2) and 22437 (v.2)). We detected 18q21.32 (chr18:57.66-57.91 hg19) deletion carriers in the UK Biobank using a threshold for the ratio of the likelihood ratio (ratio of the median likelihood ratio <−0.4 in at least 2 groups of 20 probes) followed by manual review of the genotyping fluorescence signal in the 18q21.32 region.

Within the UK Biobank, 60 unrelated (first degree or less) 18q21.32 deletion carriers were retained. We aimed to identify 50 BMI-matched UK Biobank participants for each of the 60 deletion carriers. Matched participants were retained after excluding: (1) related UK Biobank participants (third degree or less) and (2) individuals with 16p11.2 deletions, which are known CNVs associated with obesity. Participants were matched according to BMI (UK Biobank field no. 21001; ±1.0 kg m^−2^, up to BMI 35; ±2.5 kg m^−2^ for BMI 35–47 kg m^−2^; ±7 kg m^−2^ for BMI 47–53 kg m^−2^; ±10 kg m^−2^ for BMI ≥ 53 kg m^−2^), age (no. 21003; ±2.5 years, up to BMI 35; ±5 years, for BMI 35–47 kg m^−2^; ±7 years, for BMI 47–53 kg m^−2^; ±10 years, for BMI ≥ 53 kg m^−2^), sex (no. 31; identical) and self-reported ethnic background (no. 21000; identical) without replacement (that is, each control was used only once). We could not identify 50 matched participants for deletion carriers of Irish ethnicity, who were therefore excluded (*n* = 2). The final matched cohort analysis was performed on 58 deletion carriers and 2,900 matched control individuals. Participant characteristics are summarized in Supplementary Table 2. To test for early onset of obesity in 18q21.31 deletion carriers, comparative body size at age 10 (data field no. 1687*)* was compared between deletion carriers and BMI-matched controls (matched cohort analysis). Statistical analysis was performed in R v.4.1.1. using logistic regression.

### *MC4R* variant carriers in the UK Biobank identified using WES data

#### WES QC

A detailed description of WES, including experimental details, variant calling and standard quality control (QC) measures, for the UK Biobank have been reported in detail by Backman et al.^78^. We performed further QC steps using the UK Biobank RAP (https://ukbiobank.dnanexus.com). We used bcftools (v.1.15.1) to process population-level variant call format files, by first splitting multiallelic variants to subsequently filter SNPs and insertions and/or deletions (indels) for missingness. We set SNP values for participants to missing if they had a read depth <7 (<10 for indels) or genotype quality <20. We also tested each SNP for excess heterogeneity using a hypergeometric test and subsequently excluded SNPs with significant evidence (*P* < 10^−4^). Finally, we computed missingness rates for each SNP following QC and excluded SNPs with missing values in >50% of the participants.

#### Variant impact classification

We used ENSEMBL variant effect predictor, v.104 to annotate coding variants of interest within *MC4R*^79^. We used published data (summarized on the website: www.mc4r.org.uk) to classify the in vitro functional impact of variants which allowed us to define variants as LoF, wild-type like, GoF or unknown—where no functional data were available (Supplementary Table 4).

We included up to 431,579 individuals from the UK Biobank with WES data^78^. We identified individuals carrying a missense, stop gain or frameshift variant in *MC4R*. Association analyses using WES data, PGS for BMI and the comparison of *MC4R* carriers in the GOOS with noncarriers in the UK Biobank across cardiovascular and metabolic traits were conducted under UK Biobank application ID no. 44448.

### Trait preparation

All comparisons with *MC4R* variant carriers in the GOOS (up to *n* = 144) were performed with measures taken at baseline within the UK Biobank (first study visit). To ensure that only individuals with reliable BMI (field no. 21001) measures were included in the UK Biobank comparisons group, individuals were removed from comparison when they did not meet the following criteria as recommended previously^80^: individuals were removed with missing height or weight values, standing height measure >4.56 s.d. from the mean, sitting height to standing height ratio >0.75, significant difference (>4.56 s.d.) between the BMI measures calculated during the study visit (field no. 21001) and from impedance measures (field no. 23104).

The automated reading for systolic and diastolic blood pressure was used in the analysis. The average across two readings was used as a comparison. Individuals who reported taking ‘blood pressure-lowering medication’ were excluded from the comparison. For blood lipid measures (HDL, LDL, total cholesterol and TGs), individuals who reported taking ‘lipid-lowering medication’ were excluded from the comparison (*N* = 86,874). In total, we included a maximum of 336,728 individuals from the UK Biobank, who did not carry any *MC4R* variant, as described above, in the analysis as a comparator group.

### Comparison of *MC4R* carriers versus controls

We compared values for cardiometabolic traits in *MC4R* variant carriers in the GOOS with controls in the UK Biobank stratified by BMI groups: underweight (BMI < 18 kg m^−2^), normal weight (BMI 18–25 kg m^−2^), overweight (BMI 25–30 kg m^−2^), obesity (BMI 30–40 kg m^−2^) and severe obesity (BMI > 40 kg m^−2^). To test whether there was a statistically significant difference for the key traits of interest in *MC4R* variant carriers and controls, we used linear regression models to test the association of *MC4R* variant carriage with the outcomes of interest, adjusting for age, sex and BMI. Due to case–control imbalance, we calculated robust s.e.m. values through bootstrapping, resampling 100× using the R package boot (v.1.3-28). The robust s.e. was derived from the mean s.e. across these resampled groups. Statistical significance was considered at Bonferroni’s significance threshold accounting for the number of traits (*n* = 8 traits); *P* < 0.006. Linear regression models were run on scaled outcome values (mean = 0, s.d. = 1) to allow direct comparison of effects between traits and unscaled values subsequently used to allow identification of the relative effect in the appropriate units of the outcome of interest.

It should be noted that the GOOS and UK Biobank had different recruitment criteria, with the GOOS recruiting children with severe obesity and their families, whereas the UK Biobank recruited healthy middle-aged individuals. The measurement methods and assays also differed between cohorts. Therefore, we additionally conducted gene burden analyses within the UK Biobank only to assess reproducibility of our observations (see below).

### *MC4R* gene burden analyses in the UK Biobank

In gene burden analyses, we examined the association of *MC4R* variants with self-reported use at baseline of blood pressure-lowering medication, lipid-lowering medication, metformin and insulin. In addition, we defined binary traits to identify cases of hypertension and hyperlipidemia and also tested the association 626 phecodes, medical concept terms that enable harmonization of diverse medical codes across levels of healthcare, which were generated as previously described^81^. To generate phecode-based outcome variables, we mapped ICD-10, ICD-9, Read v.2, Clinical Terms v.3 term codes from self-reported or medical health records, including cancer registry, death registry, hospitalization (for example, Hospital Episode Statistics for England) and primary care (~45% of the UK Biobank population), to a set of summarized clinical entities called phecodes. Individuals with hypertension were defined based on having systolic blood pressure ≥140 mmHg and diastolic blood pressure ≥90 mmHg. Hypercholesterolemia was defined as LDL-cholesterol ≥5 or HDL-cholesterol ≤0.9 mmol l^−1^. We additionally assessed the association of a set of CVD-related outcomes with broader definitions than defined with phecodes, through grouping of ICD-10 codes: Coronary artery disease (I21.*, I22.0-1, I22.8-9, I23.0-3, I23.5-6, I23.8, I24.*, I25.1-2, I25.5-6 and I25.8-9), Myocardial infarction (MI)—ST-elevation (STEMI UK Biobank data field no. 42002), non-ST-elevation MI (NSTEMI UK Biobank data field no. 42004) and any MI (UK Biobank data field no. 42000).

To statistically assess the impact of LoF *MC4R* variants on phenotypes of interest we conducted gene burden analyses using REGENIE (v.3.2.5)^82^ via the DNAnexus Swiss Army Knife tool (v.4.9.1) in individuals of European ancestry in the UK Biobank with WES data (maximum *N* = 426,236) via a two-step procedure to account for population structure as described in detail elsewhere^82^. We used a set of high-quality genotyped variants (minor allele frequency >1%, minor allele count >100, missingness <10%, Hardy–Weinberg equilibrium *P* value >10^−15^) in the first step for individual trait predictions using the leave-one-chromosome-out scheme. These predictions were used in the second step as offset to run linear (continuous) or logistic (binary) regression models with saddle point approximation to account for case–control imbalance and rare variant associations. Each model was adjusted for age, sex, genotyping batch, assessment center and the first ten genetic principal components. For analyses adjusting additionally for BMI, BMI was also adjusted for in step 1. We also assessed the association of two previously reported common GoF variants within MC4R, Val103Ile (chr18:60372043:C:T) and Ile251Leu (chr18:60371599:T:G), using the same analytical framework used for LoF variants, considering variants in an additive model (0 = homozygous reference allele, 1 = heterozygous alternative allele and 2 = homozygous alternative allele).

Due to having a high prior of relevance of the core set of phenotypes of interest being impacted by MC4R function in the GOOS cohort, we considered significance at *P* < 0.05 for analyses of cardiovascular and glycemic traits. For phenome-wide analyses a more stringent Bonferroni’s significance threshold was considered (*P* < 1.34 × 10^−5^).

### Common PGS analyses

To compare the impact of common variation on obesity with rare *MC4R* variants in the UK Biobank, we constructed PGSs for BMI using 76 SNPs significantly associated with BMI reported in ref. ^83^ in cohorts of European ancestry. The locus at *MC4R* was excluded from the PGS construction. Alleles were constructed weighted by the effect size reported by Locke et al.^83^ and the sum of these weighed alleles used to define the PGS in each individual. Association of the PGS was tested across 626 phecodes (see above) using logistic regression models adjusted for age, sex and the first ten genetic principal components, with and without adjustment for BMI. All analyses were conducted in unrelated European subsets of the UK Biobank and a maximum of 348,303 individuals were included in the analyses.

### High-fat meal challenge

We recruited 11 heterozygous carriers of LoF *MC4R* variants and 15 adult volunteers matched for age, sex and BMI. We used the following criteria: age 18–50 years, BMI ≥ 27 kg m^−2^, weight stable for the last 3 months, no significant history of gastrointestinal, renal, hepatic or thyroid disease, average alcohol intake <2 units per day, not participating in an organized exercise program, nonsmoking, not treated with anorectic agents or medications known to affect carbohydrate and/or lipid metabolism, bile acid modifiers, other drugs affecting the gut or vitamin supplements.

Participants were resident on the National Institute for Health and Care Research (NIHR) Clinical Research Facility (Addenbrooke’s Hospital, Cambridge, UK) for the duration of the study under direct observation. They arrived the day before the meal challenge and were provided with dinner at 17:30 and snacks at 21:00, matching, respectively, 35% and 10% of their daily energy requirements calculated by the basal metabolic rate using the Schofield equation multiplied by a physical activity level of 1.25 (ref. ^84^). They were asked to refrain from strenuous scheduled physical activity and intake of alcohol, tea and coffee for 24 h. After a 10-h overnight fast, participants were cannulated and fasting blood samples were drawn at 07:45 and 08:00, for averaging of both fasting samples. The standard breakfast and the high-fat meal challenge were served at 08:00, to be finished by 08:20, then followed by 500 ml of water which had to be consumed between 08:30 and 14:00. No other food or drink was allowed until after 14:00.

The composition of the breakfast was as follows: 75 g of muesli, 220 g of semi-skimmed milk, 14 g of dried skimmed milk powder and 130 g of low-fat flavored yoghurt. The high-fat meal challenge consisted of a chocolate-flavored drink, made of 1.7 g of cocoa powder, 18 g of olive oil, 400 mg of emulsifier, 1.2 g of sweetener and 35.5 ml of water. The total content of the breakfast and the chocolate-flavored drink was 674 kcal: 26.9 g of fat (242.2 kcal), 78.2 g of carbohydrates (312.9 kcal) and 26.5 g of protein (105.9 kcal).

Plasma glucose, insulin, serum lipids, liver function tests, thyrotropin, free thyroxine and free triiodothyronine were measured using standard methods. Fasting plasma lipoproteins (average of −15 and 0 min timepoints) were isolated by sequential ultracentrifugation at 4 °C (Beckman Coulter) from 3 ml of plasma as follows: VLDL (density <1.006 g ml^−1^), intermediate lipoprotein (density 1.006–1.019 g ml^−1^) and LDL (density 1.019–1.063 g ml^−1^). After the high-fat meal challenge for breakfast, postprandial TRLs were isolated as for fasting VLDLs (Sf 20 to >400). Plasma and fractional TG, cholesterol and apoA-I concentrations were measured using standard kit methods (Horiba ABX) on an ABX Cobas MIRA autoanalyzer (Horiba ABX). Lipoprotein apoB-100 concentrations were measured by an in-house ELISA^85^. The fractions of TG and cholesterol in these lipoproteins were assessed by ultracentrifugation of the supernatant in 23 participants in the fasting state (average timepoints of −15 and 0 min) and postprandially (for 6 h) after a high-fat meal challenge for breakfast.

Data were presented as mean and s.e.m. and analyzed using Stata software package (v.15.1, StataCorp). We compared the peak (maximum) values and the time to reach this peak. Pairwise comparisons of the study phases were performed using a two-sided Student’s *t*-test when appropriate. A *P* value of 0.05 was considered significant. Cholesterol and TG levels in TRLs for each person were quantified using a trapezoidal AUC. Statistical tests were two tailed unless otherwise stated. No statistical method was used to predetermine the sample size.

Nontargeted metabolomic analysis was carried out at each timepoint of the high-fat meal challenge (time 0 min to 30 min to 60 min to 90 min to 120 min, then hourly until 360 min) for 9 of 11 individuals with MC4R deficiency and 13 of 15 control samples. The nontargeted metabolomic analysis was performed at Metabolon, Inc. All serum samples were stored at −80 °C until processed. The detailed descriptions of the platform, including sample processing, instrument configuration, data acquisition and metabolite identification and quantification, have been published previously^86,87^, with the exception that four independent ultraperformance liquid chromatography with tandem mass spectrometry methods were used.

### Reporting summary

Further information on research design is available in the Nature Portfolio Reporting Summary linked to this article.

## Online content

Any methods, additional references, Nature Portfolio reporting summaries, source data, extended data, supplementary information, acknowledgements, peer review information; details of author contributions and competing interests; and statements of data and code availability are available at 10.1038/s41591-025-03976-1.

## Supplementary information

Reporting Summary

Supplementary Tables Supplementary Tables 1–15.

## Data Availability

Anonymized clinical data are available to bona fide researchers from the corresponding author with no restrictions (I.S.F.). All data from the UK Biobank used in this study are available from the UK Biobank upon application (https://www.ukbiobank.ac.uk).
