# Supplementary material for: Obesity due to MC4R deficiency is associated with reduced cholesterol, triglycerides and cardiovascular disease risk
Source: Nat Med. 2025 Oct 16;31(12):4180–8. doi: 10.1038/s41591-025-03976-1 (PMC12705457; doi:10.1038/s41591-025-03976-1)
Supplement: Supplementary file 1 — Reporting Summary [file 41591_2025_3976_MOESM1_ESM.pdf]

nature portfolio

Corresponding author(s): Prof. T-H. Collet
Prof. I.S. Farooqi

Last updated by author(s): Aug 22, 2025

Reporting Summary

Nature Portfolio wishes to improve the reproducibility of the work that we publish. This form provides structure for consistency and transparency in reporting. For further information on Nature Portfolio policies, see our Editorial Policies and the Editorial Policy Checklist.

Statistics

For all statistical analyses, confirm that the following items are present in the figure legend, table legend, main text, or Methods section.

n/a	Confirmed	
☐	☒	The exact sample size (*n*) for each experimental group/condition, given as a discrete number and unit of measurement
☐	☒	A statement on whether measurements were taken from distinct samples or whether the same sample was measured repeatedly
☐	☒	The statistical test(s) used AND whether they are one- or two-sided<br>*Only common tests should be described solely by name; describe more complex techniques in the Methods section.*
☐	☒	A description of all covariates tested
☐	☒	A description of any assumptions or corrections, such as tests of normality and adjustment for multiple comparisons
☐	☒	A full description of the statistical parameters including central tendency (e.g. means) or other basic estimates (e.g. regression coefficient) AND variation (e.g. standard deviation) or associated estimates of uncertainty (e.g. confidence intervals)
☐	☒	For null hypothesis testing, the test statistic (e.g. *F*, *t*, *r*) with confidence intervals, effect sizes, degrees of freedom and *P* value noted<br>*Give P values as exact values whenever suitable.*
☒	☐	For Bayesian analysis, information on the choice of priors and Markov chain Monte Carlo settings
☒	☐	For hierarchical and complex designs, identification of the appropriate level for tests and full reporting of outcomes
☒	☐	Estimates of effect sizes (e.g. Cohen's *d*, Pearson's *r*), indicating how they were calculated

Our web collection on statistics for biologists contains articles on many of the points above.

Software and code

Policy information about availability of computer code

Data collection	*Provide a description of all commercial, open source and custom code used to collect the data in this study, specifying the version used OR state that no software was used.*
Data analysis	*Provide a description of all commercial, open source and custom code used to analyse the data in this study, specifying the version used OR state that no software was used.*

For manuscripts utilizing custom algorithms or software that are central to the research but not yet described in published literature, software must be made available to editors and reviewers. We strongly encourage code deposition in a community repository (e.g. GitHub). See the Nature Portfolio guidelines for submitting code & software for further information.

Data

Policy information about availability of data

All manuscripts must include a data availability statement. This statement should provide the following information, where applicable:

- Accession codes, unique identifiers, or web links for publicly available datasets
- A description of any restrictions on data availability
- For clinical datasets or third party data, please ensure that the statement adheres to our policy

GOOS WES data are accessible from the European Genome-Phenome Archive-EGA:EGAS00001000124. Access to the UK Biobank genotype and phenotype data open to all approved health researchers, accessible through https://www.ukbiobank.ac.uk. Requests for anonymised human data may be addressed to the

corresponding author (I.S.F.). Limitations on clinical data (which have to be anonymised) are designed to protect and respect patient and participant's confidentiality.

Research involving human participants, their data, or biological material

Policy information about studies with human participants or human data. See also policy information about sex, gender (identity/presentation), and sexual orientation and race, ethnicity and racism.

Reporting on sex and gender	Sex was assigned by clinical examination by physicians.
Reporting on race, ethnicity, or other socially relevant groupings	Ethnicity was self-reported by participants.
Population characteristics	Participants recruited from the Genetics of Obesity Study (www.goos.org.uk) were referred by their physicians based on the following criteria:<br>(1) Early onset obesity (before age of 10 years), and<br>(2) Severe obesity as defined by a BMI ≥ 3 standard deviation score
Recruitment	Participants were referred to the Genetics of Obesity Study by their physicians if they satisfied the criteria listed above, irrespective of their physical location. The travel costs of participants were reimbursed. No other reimbursement for taking part was provided.
Ethics oversight	All studies were approved by the Multi-Regional Ethics Committee and the Cambridge Local Research Ethics Committee (MREC 97/21 and REC number 03/103). All participants, or their legal guardian for those aged under 16, provided written consent for all assessments; in addition, participants under the age of 16 provided oral assent.

Note that full information on the approval of the study protocol must also be provided in the manuscript.

Field-specific reporting

Please select the one below that is the best fit for your research. If you are not sure, read the appropriate sections before making your selection.

☒ Life sciences ☐ Behavioural & social sciences ☐ Ecological, evolutionary & environmental sciences

For a reference copy of the document with all sections, see nature.com/documents/nr-reporting-summary-flat.pdf

Life sciences study design

All studies must disclose on these points even when the disclosure is negative.

Sample size	All participants harbouring rare variants of MC4R were included.
Data exclusions	No data was excluded.
Replication	Rare variants identified by exome sequencing were validated by Sanger sequencing. All mutations were validated using this methods.
Randomization	No randomization was conducted in this observational cohort.
Blinding	Analyses were conducted without blinding of investigators.

Reporting for specific materials, systems and methods

We require information from authors about some types of materials, experimental systems and methods used in many studies. Here, indicate whether each material, system or method listed is relevant to your study. If you are not sure if a list item applies to your research, read the appropriate section before selecting a response.

Materials & experimental systems

n/a	Involved in the study
☒	☐ Antibodies
☒	☐ Eukaryotic cell lines
☒	☐ Palaeontology and archaeology
☒	☐ Animals and other organisms
☒	☐ Clinical data
☒	☐ Dual use research of concern
☒	☐ Plants

Methods

n/a	Involved in the study
☒	☐ ChIP-seq
☒	☐ Flow cytometry
☒	☐ MRI-based neuroimaging

Plants

Seed stocks	n/a
Novel plant genotypes	n/a
Authentication	n/a
